# Supplementary material for: Cell loss disrupts mechanical homeostasis to drive retinal pigment epithelium ageing-like phenotype in vitro
Source: Nat Commun. 2026 Apr 8;17:3404. doi: 10.1038/s41467-026-71493-x (PMC13068956; doi:10.1038/s41467-026-71493-x)
Supplement: Supplementary file 1 — Supplementary Information [file 41467_2026_71493_MOESM1_ESM.pdf]

Supplementary information for

Cell loss disrupts mechanical homeostasis to drive retinal pigment epithelium ageing-like phenotype *in vitro*

Teodora Piskova, Aleksandra N. Kozyrina, Giedrė Astrauskaitė, Mohamed Elsafi Mabrouk, Sebastian Schepl, Stacy Lok Sze Yam, Ragul Ravithas, Wolfgang Wagner, Massimo Vassalli and Jacopo Di Russo

Contents

Supplementary Figures 1 – 9	Page 1-9
Uncropped Immunoblots Supplementary Figure 6C	Page 10
References	Page 11

Supplementary Figures

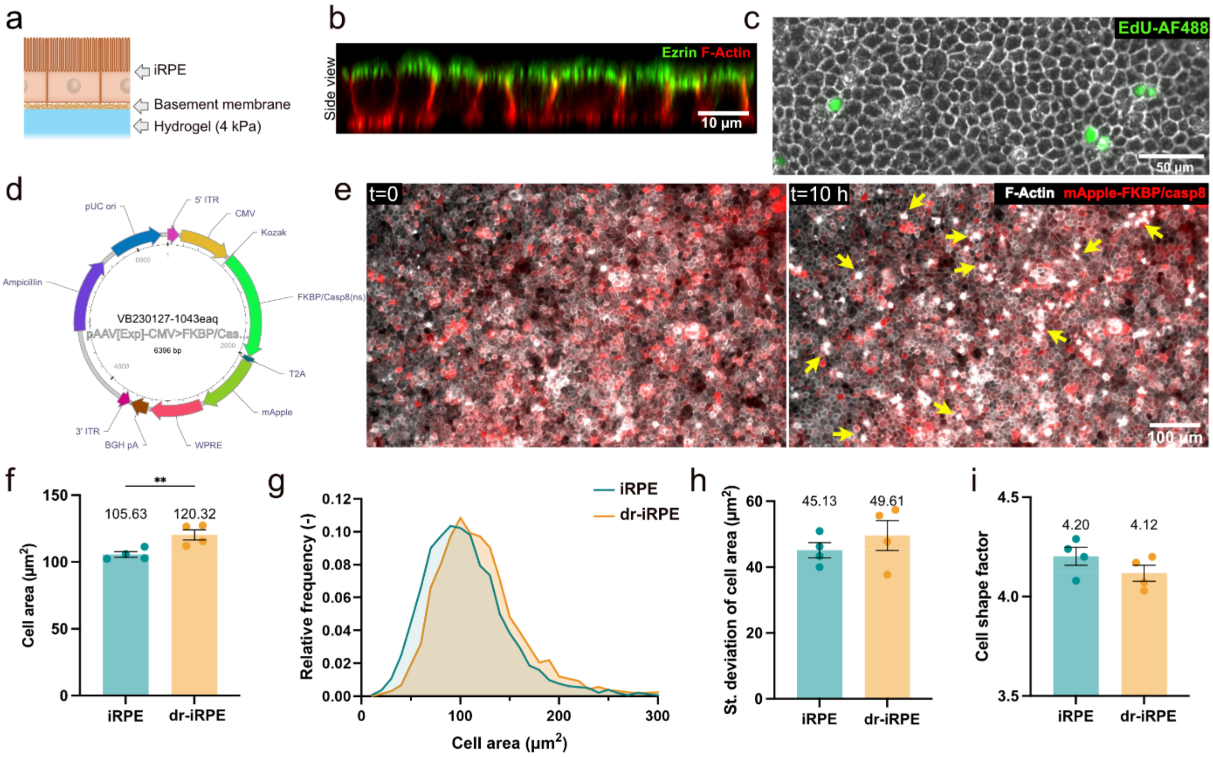

Supplementary figure 1: The iRPE and dr-iRPE model and cell morphometric characterisation. a Schematics of iRPE cell culture system displaying the monolayer obtained on 4 kPa hydrogels functionalised with basement membrane mimicking coating. **b** Cross-section of iRPE after 20 days of culture labelled for F-actin (red) and ezrin (green) showing characteristic apicobasal polarisation. **c** Representative micrograph of proliferation assay showing iRPE cells in brightfield and proliferating cell nuclei labelled by EdU-AF488 (green). **d** Map of plasmid used for the expression of pro-apoptotic FKBP-casp8 protein and delivered to iRPE cells by AAV5. Plasmid map was generated using VectorBuilder. **e** Representative micrographs of SiR-actin labelled iRPE (white) expressing mApple as fluorescent reporter (red) at the timepoint of AP20187 addition and 10 h afterwards. Extrusion events of apoptotic cells at the latter point are indicated with yellow arrows. **f** Quantification of average cell area (n=4 independent experiments, p=0.0087). **g** Relative frequency distribution of cell areas (from 3263 (iRPE) and 2821 (dr-iRPE) cells from n=5 independent experiments). **h** Standard deviation of cell areas within an experiment (n=4 independent experiments). **i** Average cell shape factor (n=4 independent experiments). Datapoints represent average values ± SEM per experiment. Statistical significance was tested using a two-sided paired t-test, where **: p<0.01. Micrographs in b, c and e are representative of four independent experiments with similar results. Source data are provided as a Source Data file.

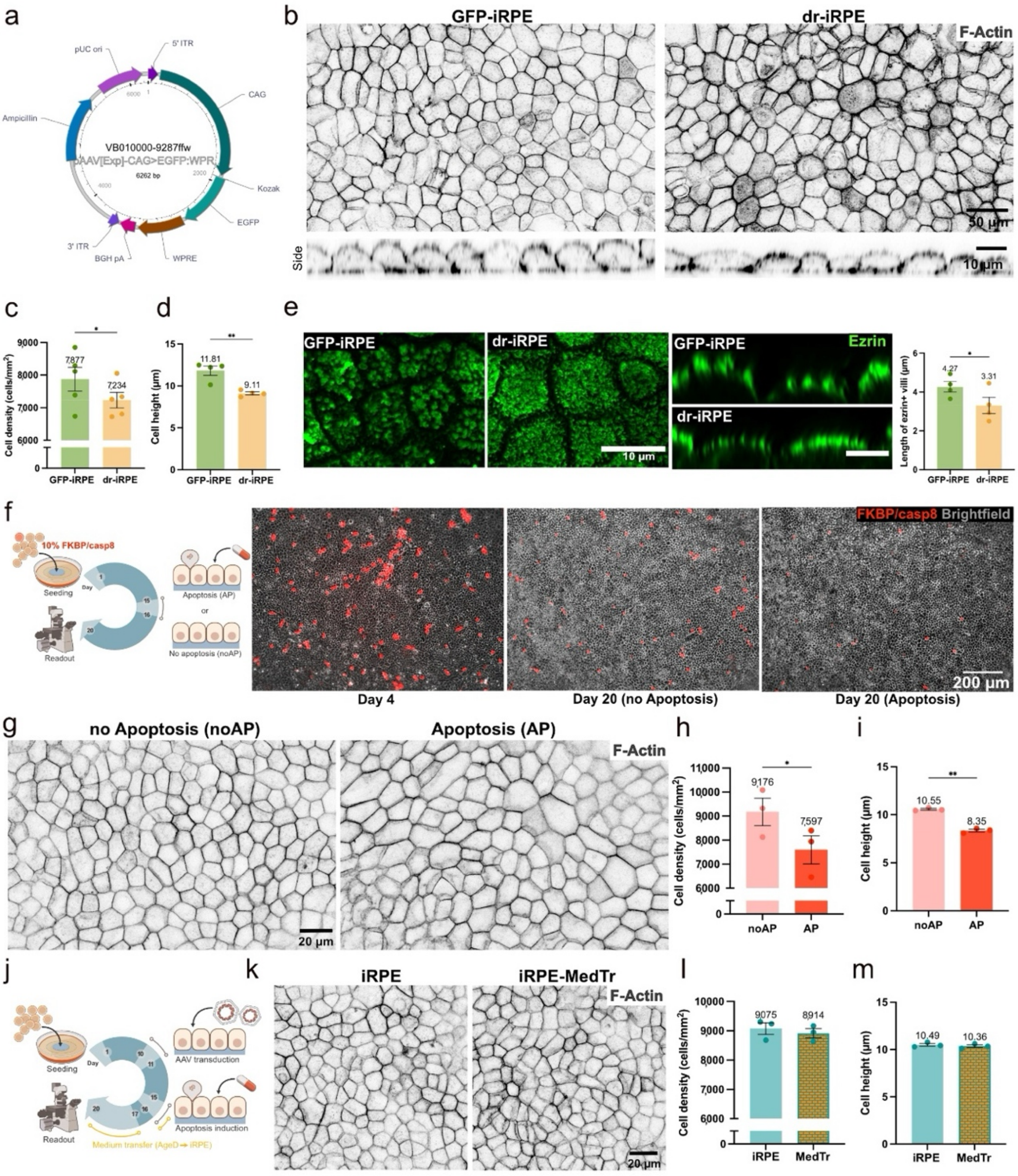

Supplementary figure 2: Controls for influence of virus, recombinant FKBP-casp8 protein expression and debris released into medium. a Vector map of plasmid used as viral control, which promotes the expression of GFP. Plasmid map was generated using VectorBuilder. **b** Representative micrographs of F-actin in GFP-iRPE compared to dr-iRPE (top) and cross sections (down). **c** Quantification of average cell density for GFP-iRPE (n=5 independent experiments, p=0.0253). **d** Quantification of cell height for GFP-iRPE (n=4 independent experiments, p=0.0083). **e** Representative micrographs showing ezrin as maximum intensity projection in top (left) and side views (middle) and the quantification of ezrin-positive microvilli length (right, n=4 independent experiments, p=0.0286). **f** Experimental schematics of mixed population control. The micrographs show representative monolayers of mApple-positive transduced cells within the naïve cells at day 4 and at day 20 (the readout day). **g** Representative micrographs of F-actin labelled mixed population iRPE without apoptosis induction (noAP) and after induction of apoptosis (AP). **h** Quantification of average cell density for the mixed population control (n=3 independent experiments, p=0.0465). **i** Quantification of cell height for the mixed population control (n=3 independent experiments, p=0.0035). **j** Experimental schematics of medium-transfer control **k** Representative micrographs of F-actin labelled control monolayers vs. monolayers subjected to medium transfer. **l** Quantification of average cell density for the medium transfer control (n=3 independent experiments). **m** Quantification of cell height for the medium transfer control (n=3 independent experiments). Datapoints represent average values per experiment ± SEM. Statistical significance was tested using a two-sided paired t-test, where *: p<0.05, **: p<0.01. Representative micrographs in b, f, g and k are representative of three independent experiments with similar results. Source data are provided as a Source Data file. Illustrations in f and j contains NIAID NIH BioArt elements[1–7] .

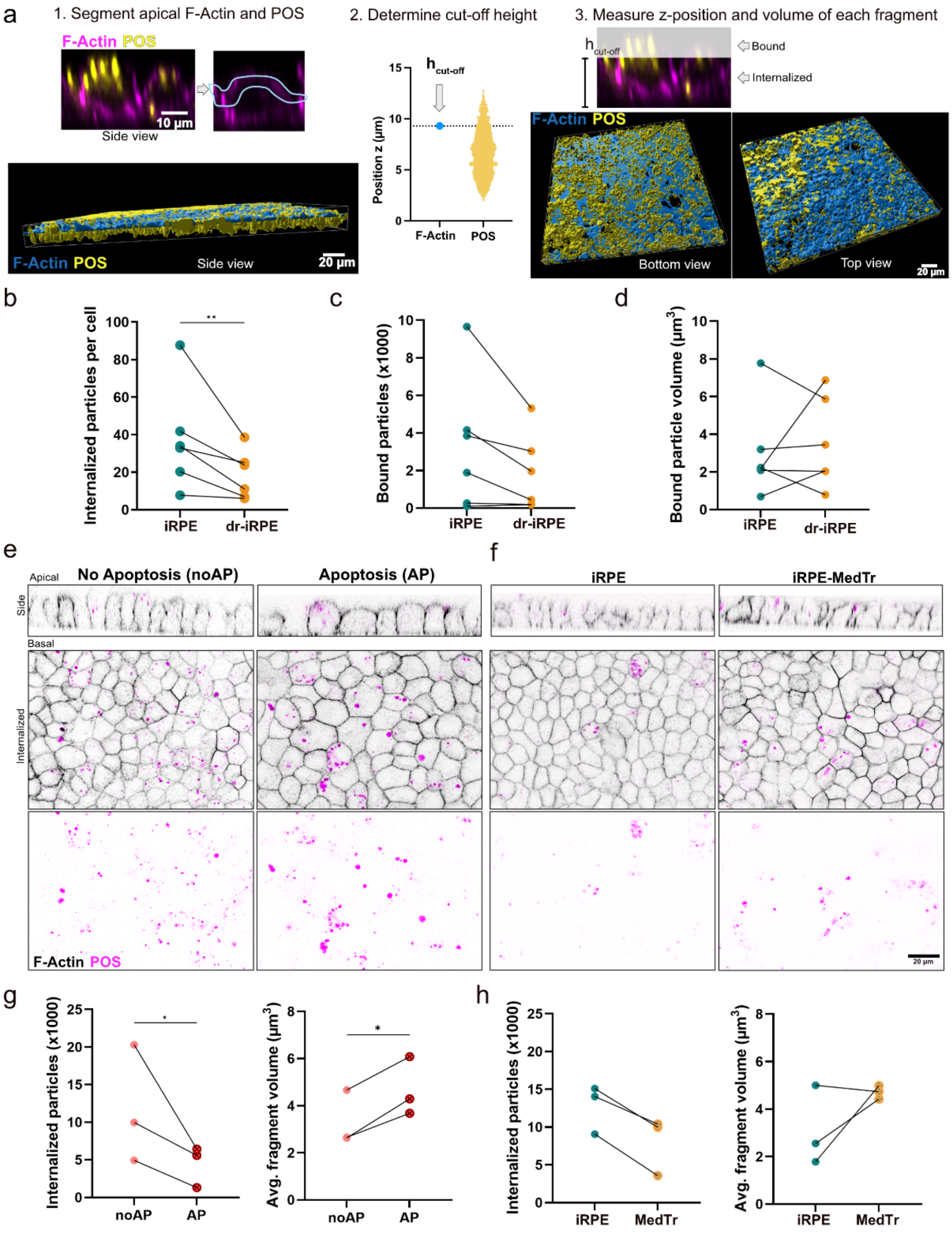

Supplementary figure 3: POS internalisation assay. a Workflow for 3D segmentation, cut-off height determination and classification into 'bound' vs 'internalised' particles. **b** Quantification of number of internalised POS particles normalised to cell number (n=6 independent experiments, p=0.0071). **c** Quantification of bound POS particles number in the field of view (n=6 independent experiments). **d** Quantification of average bound POS particle volume (n=6 independent experiments). **e** Representative micrographs of iRPE at t = 4 h of POS internalisation assay as orthogonal cross-sections (up) or as single optical slices in the middle of cells (lower two panels) for the "mixed" control. **f** Representative micrographs of iRPE at t = 4 h of POS internalisation assay as orthogonal cross-sections (up) or as single optical slices in the middle of cells (lower two panels) for the medium-transfer control (MedTr). **g** Quantification of POS fragments internalised within the time of the assay per field of view (left, p=0.0457) and quantification of the average volume of internalised fragments (right, p=0.0328) for the "mixed" population control (n=3 independent experiments). **h** Quantification of POS fragments internalised within the time of the assay per field of view (left) and quantification of the average volume of internalised fragments (right) for the medium-transfer control (n=3 independent experiments). Datapoints represent average values per experiment. Statistical significance was tested using a two-sided paired t-test, where *: p<0.05, **: p<0.01. Micrographs in e and f are representative of three independent experiments with similar results. Source data are provided as a Source Data file.

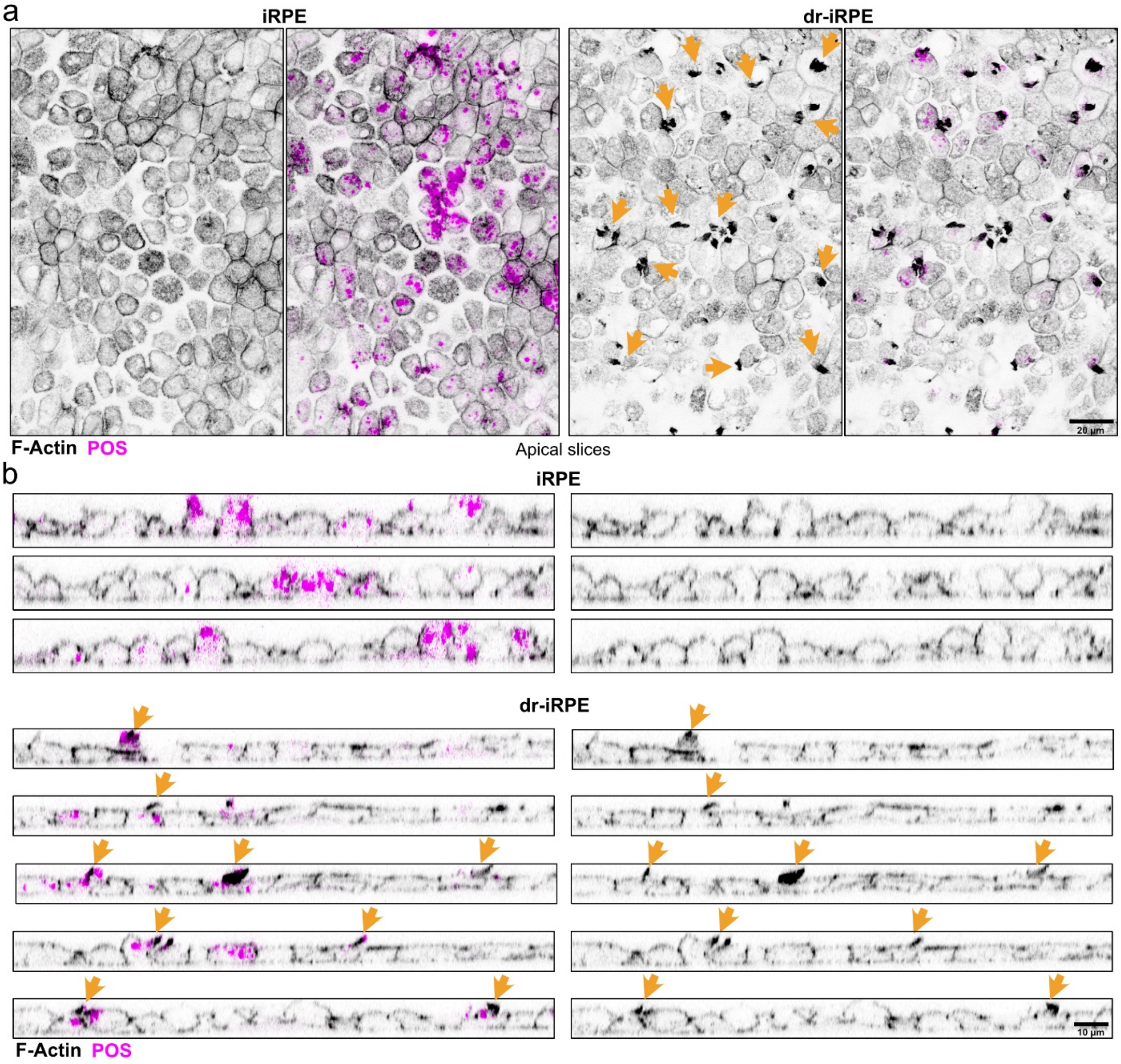

Supplementary figure 4: Differential apical actomyosin remodelling between iRPE and dr-iRPE during POS internalisation assay. a Representative micrographs of the apical side of monolayers during internalisation showing F-actin and photoreceptors. Arrows point to the presence of protruding actin-rich structures in dr-iRPE. **b** Representative orthogonal cross sections of iRPE and dr-iRPE monolayers during internalisation showing F-actin and FITC-labelled POS. Arrows point to actin-rich structures. Micrographs are representative of three independent experiments with similar results.

a

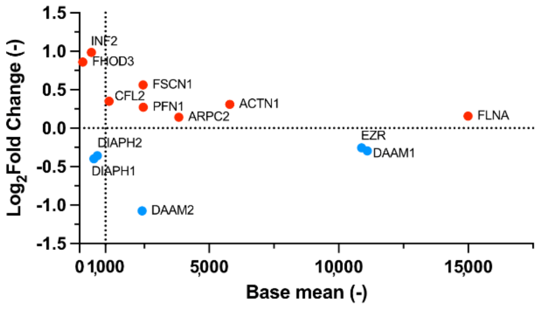

b

Overlapping DEGs with reported age-related changes in humans

Gene symbol	Protein name	Direction change
	Cai H. et al. 2012 (DOI:10.3389/fnagi.2012.00008)	
DKK1	dickkopf WNT signaling pathway inhibitor 1(DKK1)	up
LAMA3	laminin subunit alpha 3(LAMA3)	up
TYMS	thymidylate synthetase(TYMS)	up
PLEKHG4	pleckstrin homology and RhoGEF domain containing G4(PLEKHG4)	down
SCN2B	sodium voltage-gated channel beta subunit 2(SCN2B)	down
	Butler J. M. et al. 2021 (DOI: 10.1111/jcmm.16569)	
AKAP6	A-kinase anchoring protein 6(AKAP6)	up
BMP8B	bone morphogenetic protein 8b(BMP8B)	up
CCDC51	coiled-coil domain containing 51(CCDC51)	up
CTSV	cathepsin V(CTSV)	up
DUSP6	dual specificity phosphatase 6(DUSP6)	up
KL	klotho(KL)	up
SLC6A20	solute carrier family 6 member 20(SLC6A20)	up
TNFRSF10C	TNF receptor superfamily member 10c(TNFRSF10C)	up
TRPV4	transient receptor potential cation channel subfamily V member 4(TRPV4)	up
CPAMD8	C3 and PZP like alpha-2-macroglobulin domain containing 8(CPAMD8)	down
CSRP2	cysteine and glycine rich protein 2(CSRP2)	down
DCT	dopachrome tautomerase(DCT)	down
GAS1	growth arrest specific 1(GAS1)	down
TMEM150C	transmembrane protein 150C (TMEM150C)	down

c d

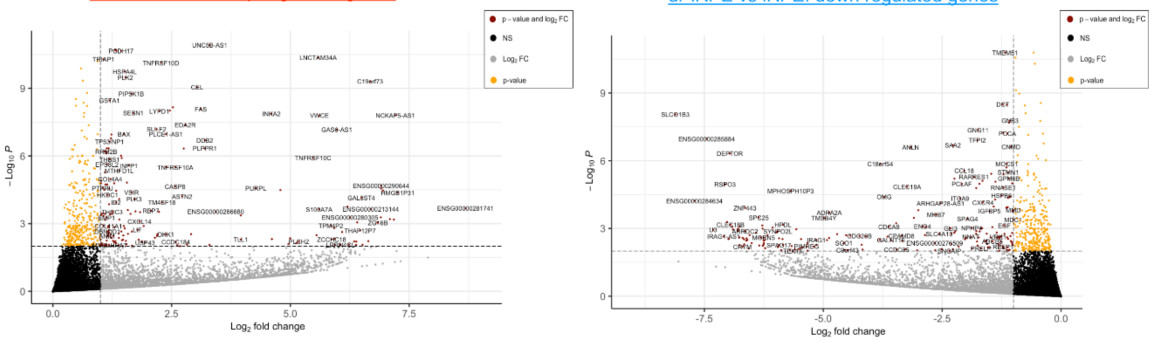

Supplementary figure 5: Density reduced iRPE shows a differential gene expression from iRPE, which is partly overlapping with ageing patients. a Base mean vs Log2Fold change for significantly differentially expressed actin-associated genes in dr-iRPE vs iRPE (n=4 independent experiments). **b** Differentially expressed genes (DEGs) overlapping with the results from studies on ageing human RPE from Cai H. et al. [8] and Butler J. M. et al. [9]. Gene names are labelled in blue or red for downregulated or upregulated, respectively. **c** Volcano plot of upregulated DEGs between dr-iRPE and iRPE. **d** Volcano plot of downregulated DEGs between dr-iRPE and iRPE. Cut-off lines were drawn at log2fold=1, corresponding to a fold change of two, and at log10P = -2, corresponding to a p of 0.001 and padj of 0.1, which we set as cut-off significance value for DEGs. Genes are assigned to groups based on their log2fold and log10fold values as highlighted in the legend. Source data are provided as a Source Data file.

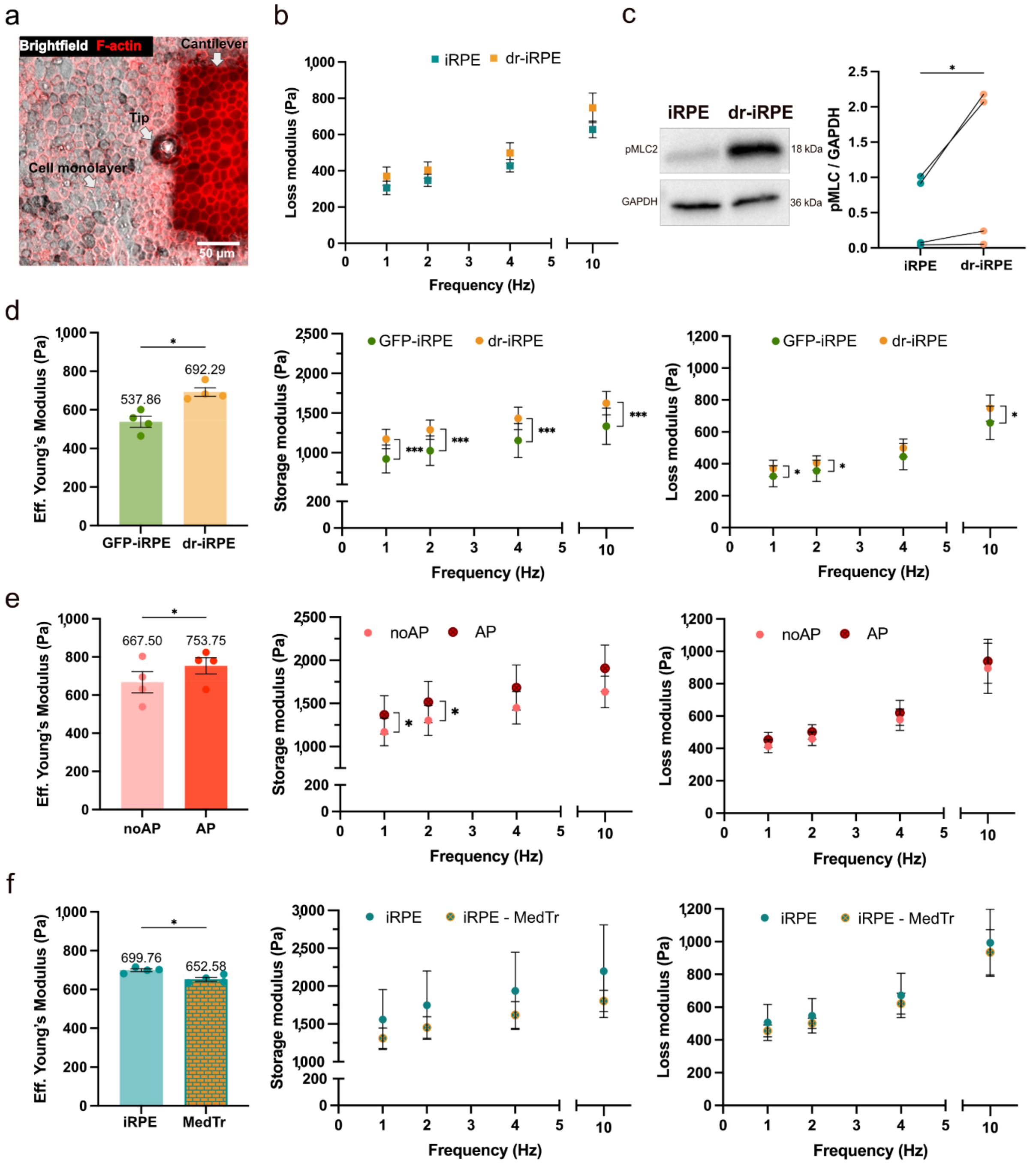

Supplementary figure 6: Biomechanical characterisation of iRPE conditions. a Experimental setup for performing nanoindentation. The micrograph shows a top view of SiR-actin labelled cell monolayer and cantilever with tip. **b** Average loss modulus of iRPE and dr-iRPE (n=4 independent experiments). **c** Immunoblot of pMLC2 and GAPDH as a housekeeping protein and quantification of total pMLC2 protein from immunoblots, normalised to GAPDH (n=4 independent experiments, p=0.0226). **d** Average Effective Young's modulus (p=0.0340), storage (p=0.0048 (1 Hz), 0.0085 (2 Hz), 0.0088 (4 Hz), 0.0089 (10 Hz)) and loss moduli (p=0.0140 (1 Hz), 0.0362 (2 Hz), 0.0379 (10 Hz)) for virus control (n=4 independent experiments). **e** Average Effective Young's modulus ((p=0.0476), storage ((p=0.0375 (1 Hz), 0.0464 (2 Hz)) and loss moduli for treated (noAP) and untreated (AP) mixed population iRPE (n=4 independent experiments). **f** Average Effective Young's modulus (p= 0.0316), storage and loss moduli for iRPE and conditioned medium treated iRPE (MedTr-iRPE) (n=4 independent experiments). Datapoints represent average values ± SEM per experiment. Statistical significance was tested using a two-sided paired t-test, where *: p<0.05 and ***: p<0.001. Source data are provided as a Source Data file.

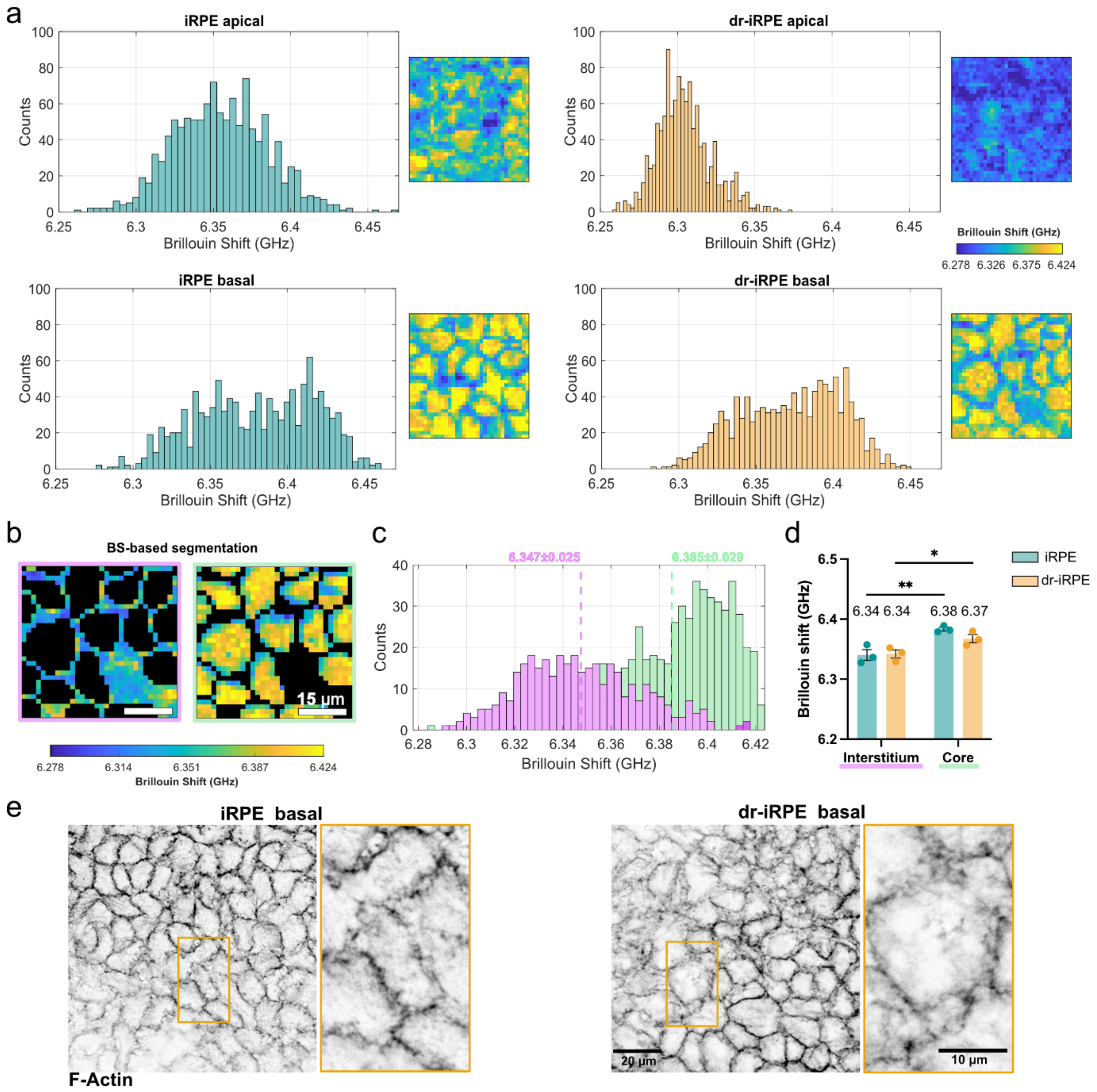

Supplementary figure 7: Distribution of Brillouin shift values in apical and basal layers of iRPE and density reduced iRPE and segmentation of Brillouin shift values from basal maps. a Representative histograms of iRPE and dr-iRPE basal and apical Brillouin shift values acquired from 50 x 50 µm regions of interest and the respective plots. **b** Representative plots of segmented Brillouin shift maps from basal scans with two population modes defined by Cellpose segmentation (see Methods). **c** Representative histogram of the plots shown in B illustrating the identified populations. **d** Quantification of Brillouin shift population modes named as “Interstitium” and “Core” in basal iRPE and dr-iRPE monolayers (n=3 independent experiments, p= 0.0021 (Int-iRPE vs Core-iRPE), 0.0134 (Int-dr-iRPE vs Core-dr-iRPE)). **e** Representative F-Actin micrographs of basal iRPE and dr-iRPE monolayers with annotated regions enlarged. Datapoints in d represent average values per experiment ± SEM. Statistical significance was tested using a one-way ANOVA, where *: $p<0.05$ and **: $p<0.01$. Micrographs in e are representative of four independent experiments with similar results. Source data are provided as a Source Data file.

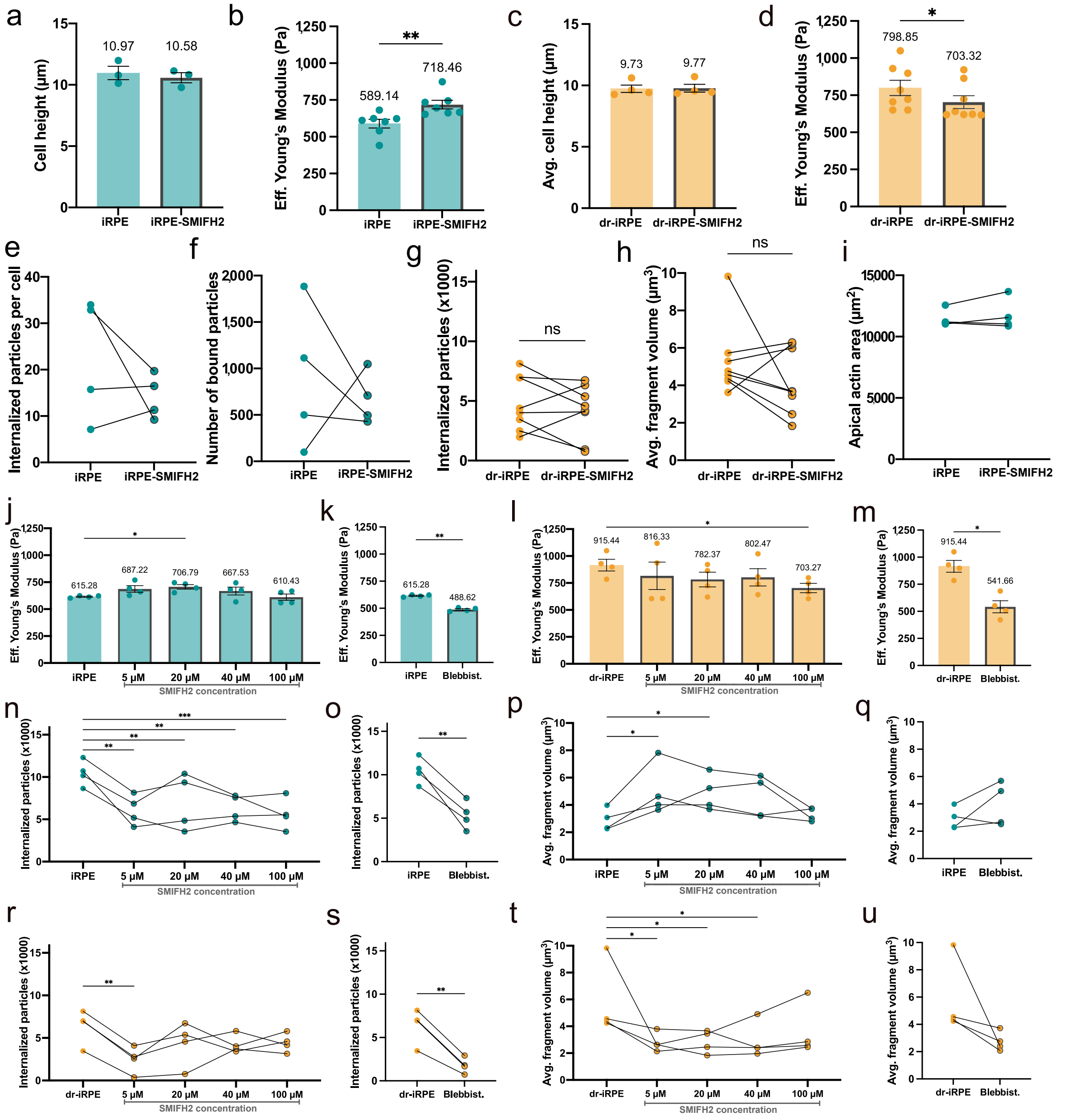

Supplementary figure 8: Linear nucleator inhibition with SMIFH2 during photoreceptor phagocytosis. a Cell height of iRPE ±SMIFH2 (n=3). **b** Effective Young's Modulus of iRPE ±SMIFH2 (n=7). **c** Cell height of dr-iRPE ±SMIFH2 (n=4). **d** Effective Young's Modulus of dr-iRPE ±SMIFH2 (n=8). **e** Internalised POS particles normalised to cell number in iRPE ±SMIFH2 (n=4). **f** Bound POS particles per field of view in iRPE ±SMIFH2 (n=4). **g** Internalised particles in dr-iRPE ±SMIFH2 (n=8). **h** Average internalised POS particle in dr-iRPE ±SMIFH2 (n=8). **i** Apical actin area per field of view during POS internalisation in iRPE ±SMIFH2 (n=4). **j** Effective Young's Modulus of iRPE monolayers after 1 h SMIFH2 treatment (5, 20, 40 or 100 µM; n=4). **k** Effective Young's Modulus of iRPE monolayers after 1h of 5 µM blebbistatin treatment (n=4). **l** Effective Young's Modulus of dr-iRPE monolayers after 1 h SMIFH2 treatment (5, 20, 40 or 100 µM; n=4). **m** Effective Young's Modulus of dr-iRPE monolayers after 1 h of 5 µM blebbistatin treatment (n=4). **n** Internalized POS particle number in iRPE after SMIFH2 treatment (5, 20, 40 or 100 µM; n=4). **o** Internalized POS particle number in iRPE after 5 µM blebbistatin treatment (n=4). **p** Average internalized POS particle volume in iRPE after SMIFH2 treatment (5, 20, 40 or 100 µM; n=4). **q** Average internalized POS particle volume in iRPE after 5 µM blebbistatin treatment (n=4). **r** Internalized POS particle number in dr-iRPE after SMIFH2 treatment (5, 20, 40 or 100 µM; n=4). **s** Internalized POS particles number of dr-iRPE after 5 µM blebbistatin treatment (n=4). **t** Average internalized POS particle volume in dr-iRPE after SMIFH2 treatment (5, 20, 40 or 100 µM; n=4). **u** Average internalized POS particle volume in dr-iRPE after 5 µM blebbistatin treatment. Datapoints represent average values (± SEM for bar graphs) per n independent experiments. Statistical significance in a - d, i, k and m was tested using two-sided paired t-test; in e - h, o, q, s and u – using two-sided ratio paired t-test; in j, l, n, p, r and t – using One-way ANOVA with multiple comparisons. *: $p<0.05$ and **: $p<0.01$. Source data and p-values are provided as a Source Data file.

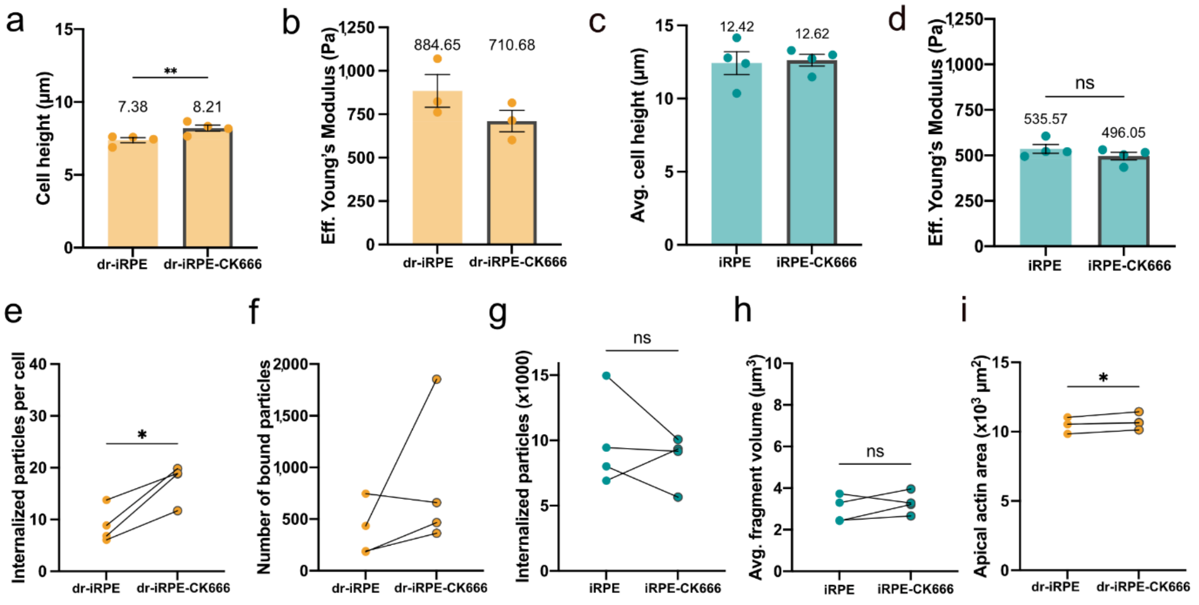

Supplementary figure 9: Branched nucleator inhibition with CK666 during photoreceptor phagocytosis. a Quantification of cell height for dr-iRPE vs CK666-treated dr-iRPE (n=4 independent experiments, p=0.0019). **b** Average Effective Young's Modulus, derived from 2.5 µm nanoindentation with a 20 µm spherical tip for dr-iRPE vs CK666-treated dr-iRPE (n=3 independent experiments). **c** Quantification of cell height for iRPE vs CK666-treated iRPE (n=4 independent experiments). **d** Average Effective Young's Modulus, derived from 2.5 µm nanoindentation with a 20 µm spherical tip for iRPE vs CK666-treated iRPE (n=4 independent experiments). **e** Quantification of the number of internalised photoreceptor particles normalised to cell number for dr-iRPE vs CK666-treated dr-iRPE monolayers (n=4 independent experiments, p=0.0177). **f** Quantification of the number of bound particles in the field of view for dr-iRPE vs CK666-treated dr-iRPE (n=4 independent experiments). **g** Number of internalized POS particles of iRPE vs CK666-treated iRPE (n=4 independent experiments). **h** Average volume of internalized particles of iRPE vs CK666-treated iRPE (n=4). **i** Quantification of apical actin area per field of view of monolayers during internalisation in dr-iRPE vs CK666-treated dr-iRPE (n=3 independent experiments, p=0.0455). Datapoints represent average values (± SEM in case of bar graphs) per experiment. Statistical significance in a – d and i was tested using a two-sided paired t-test, where *: p<0.05 and **: p<0.01. Statistical significance in e - h was tested using a two-sided ratio paired t-test, where *: p<0.05. Source data are provided as a Source Data file.

Uncropped Immunoblots Supplementary Figure 6C

<table>
<tr><td>S. Figure 6 C: pMLC immunoblot</td><td>S. Figure 6 C: Corresponding marker</td></tr>
<tr><td colspan="2">Note: Blot in the figure is represented in inverted grayscale</td></tr>
<tr><td>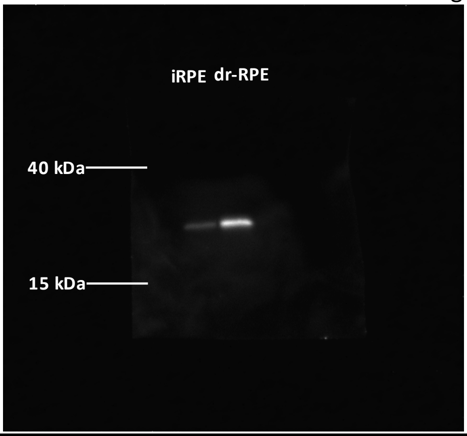
</td><td>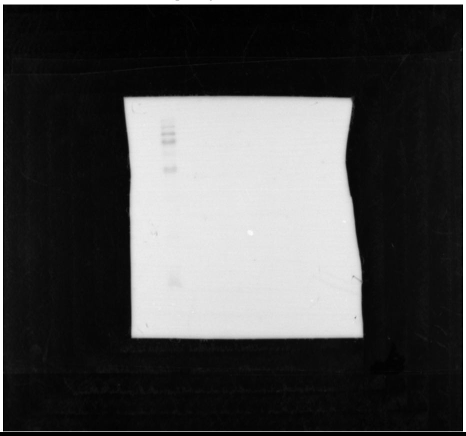</td></tr>
<tr><td>S. Figure 6 C: GAPDH immunoblot</td><td>S. Figure 6 C: Corresponding marker</td></tr>
<tr><td colspan="2">Note: Blot in the figure is represented in inverted grayscale</td></tr>
<tr><td>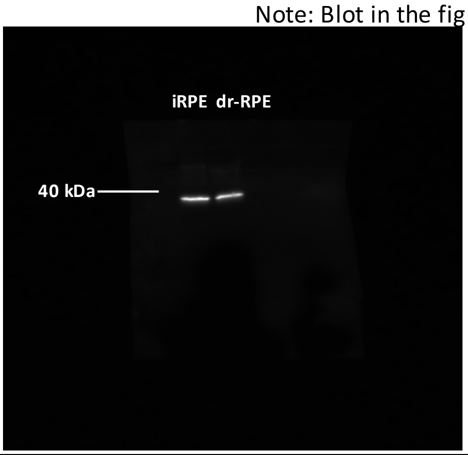
</td><td>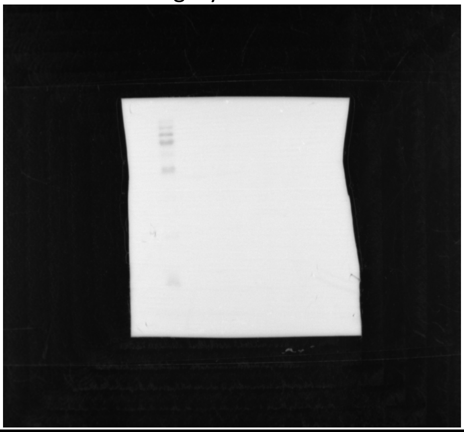</td></tr>
</table>

References

1. NIAID Visual & Medical Arts. Petri Dish. NIAID NIH BIOART Source. Bioart.Niaid.Nih.Gov/Bioart/404. (2024).

2. NIAID Visual & Medical Arts. T Cell. NIAID NIH BIOART Source. Bioart.Niaid.Nih.Gov/Bioart/509. (2024).

3. NIAID Visual & Medical Arts. Plasmid. NIAID NIH BIOART Source. Bioart.Niaid.Nih.Gov/Bioart/411. (2024).

4. NIAID Visual & Medical Arts. Enveloped Virus. NIAID NIH BIOART Source. Bioart.Niaid.Nih.Gov/Bioart/139. (2024).

5. NIAID Visual & Medical Arts. Basal Cell Brush. NIAID NIH BIOART Source. Bioart.Niaid.Nih.Gov/Bioart/45. (2024).

6. NIAID Visual & Medical Arts. Super Resolution Fluorescence Microscopy. NIAID NIH BIOART Source. Bioart.Niaid.Nih.Gov/Bioart/503. (2024).

7. NIAID Visual & Medical Arts. Pill. NIAID NIH BIOART Source. Bioart.Niaid.Nih.Gov/Bioart/407. (2024).

8. Cai, H., Fields, M. A., Hoshino, R. & Priore, L. V. D. Effects of Aging and Anatomic Location on Gene Expression in Human Retina. *Front. Aging Neurosci.* **4**, 8 (2012).

9. Butler, J. M., Supharattanasitthi, W., Yang, Y. C. & Paraoan, L. RNA-seq analysis of ageing human retinal pigment epithelium: Unexpected up-regulation of visual cycle gene transcription. *J. Cell. Mol. Med.* **25**, 5572–5585 (2021).
