# Supplementary material for: Cell loss disrupts mechanical homeostasis to drive retinal pigment epithelium ageing-like phenotype in vitro
Source: Nat Commun. 2026 Apr 8;17:3404. doi: 10.1038/s41467-026-71493-x (PMC13068956; doi:10.1038/s41467-026-71493-x)
Supplement: Supplementary file 2 — Description of Additional Supplementary Files [file 41467_2026_71493_MOESM2_ESM.pdf]

Description of Additional Supplementary Files

File Name: Supplementary Movie 1

Description: On-demand activation of FKBP/casp8 by AP20187 leads to monolayer-scale extrusion events. Live-iRPE monolayer time-lapse expressing FKBP/casp8 (mApple-positive red cells) and labelled with actin dye SiR-Actin (white). Upon the addition of the AP20187 compound, cells are extruded due to apoptosis induction. Extrusion events include neighbouring cells adopting a characteristic rosette-like morphology, while the dying cell is extruded out of the monolayer, forming a bright blob.

File Name: Supplementary Movie 2

Description: Live-cell time-lapse of iRPE monolayer during nanoindentation. The SiR-Actin labelled iRPE monolayer is probed by nanoindentation in a matrix scan (left). A zoom-in view (right) reveals the junctional deformation occurring during the measurement.
