# Supplementary material for: Cell loss disrupts mechanical homeostasis to drive retinal pigment epithelium ageing-like phenotype in vitro
Source: Nat Commun. 2026 Apr 8;17:3404. doi: 10.1038/s41467-026-71493-x (PMC13068956; doi:10.1038/s41467-026-71493-x)
Supplement: Supplementary file 5 — Reporting Summary [file 41467_2026_71493_MOESM5_ESM.pdf]

nature portfolio

Corresponding author(s): Jacopo Di Russo

Last updated by author(s): Mar 6, 2026

Reporting Summary

Nature Portfolio wishes to improve the reproducibility of the work that we publish. This form provides structure for consistency and transparency in reporting. For further information on Nature Portfolio policies, see our Editorial Policies and the Editorial Policy Checklist.

Statistics

For all statistical analyses, confirm that the following items are present in the figure legend, table legend, main text, or Methods section.

n/a	Confirmed	
☐	☒	The exact sample size (*n*) for each experimental group/condition, given as a discrete number and unit of measurement
☐	☒	A statement on whether measurements were taken from distinct samples or whether the same sample was measured repeatedly
☐	☒	The statistical test(s) used AND whether they are one- or two-sided<br>*Only common tests should be described solely by name; describe more complex techniques in the Methods section.*
☒	☐	A description of all covariates tested
☐	☒	A description of any assumptions or corrections, such as tests of normality and adjustment for multiple comparisons
☐	☒	A full description of the statistical parameters including central tendency (e.g. means) or other basic estimates (e.g. regression coefficient) AND variation (e.g. standard deviation) or associated estimates of uncertainty (e.g. confidence intervals)
☐	☒	For null hypothesis testing, the test statistic (e.g. *F*, *t*, *r*) with confidence intervals, effect sizes, degrees of freedom and *P* value noted<br>*Give P values as exact values whenever suitable.*
☒	☐	For Bayesian analysis, information on the choice of priors and Markov chain Monte Carlo settings
☒	☐	For hierarchical and complex designs, identification of the appropriate level for tests and full reporting of outcomes
☒	☐	Estimates of effect sizes (e.g. Cohen's *d*, Pearson's *r*), indicating how they were calculated

Our web collection on statistics for biologists contains articles on many of the points above.

Software and code

Policy information about availability of computer code

Data collection	ZEN black 2.1 SP3, Carl Zeiss; ZEN 3.0 blue edition, Carl Zeiss; Piuma V3.5.0, Optics11 Life; FusionCapt Advance, Vilber Lourmat Sté
Data analysis	GraphPad Prism 10, GraphPad Software, Inc.; ImageJ (Fiji distribution), National Institutes of Health; MATLAB & Simulink R2022b, MathWorks Inc.; Nanoindenter DataViewer v2.6.0, Optics11 Life; Prova v.0.9.0 Dev, Optics11 Life; R v4.4.3, R Foundation for Statistical Computing; Cellpose v.3.0.11, open source; Python v3.10.15, Python Software Foundation; nf-core/rnaseq v3.12.0, open source; Nextflow v23.04.1, Seqera Labs; Imaris Image Analysis Software v10.2.0, Oxford Instruments; LightMachinery LabView software, LightMachinery. Matlab script for "peak fitting" is available on https://github.com/GiedreAstra/Example_E3_zscan_RPE.git.

For manuscripts utilizing custom algorithms or software that are central to the research but not yet described in published literature, software must be made available to editors and reviewers. We strongly encourage code deposition in a community repository (e.g. GitHub). See the Nature Portfolio guidelines for submitting code & software for further information.

Data

Policy information about availability of data

All manuscripts must include a data availability statement. This statement should provide the following information, where applicable:

- Accession codes, unique identifiers, or web links for publicly available datasets
- A description of any restrictions on data availability
- For clinical datasets or third party data, please ensure that the statement adheres to our policy

All data are available in the main text or the supplementary materials. Furthermore, the complete data set of RNA bulk sequencing is available on the Gene

Expression Omnibus repository (GEO accession number GSE297557).

Research involving human participants, their data, or biological material

Policy information about studies with human participants or human data. See also policy information about sex, gender (identity/presentation), and sexual orientation and race, ethnicity and racism.

Reporting on sex and gender	HiPSCs-derived RPE cells used in this study were obtained from a male donor. The sex of the donor has no anticipated relevance to the study's objectives or outcomes, as the experiments did not involve sex-specific variables or analyses.
Reporting on race, ethnicity, or other socially relevant groupings	HiPSCs-derived RPE cells used in this study were obtained from a caucasian donor. The race of the donor has no anticipated relevance to the study's objectives or outcomes, as the experiments did not involve race-specific variables or analyses.
Population characteristics	n/a
Recruitment	n/a
Ethics oversight	n/a

Note that full information on the approval of the study protocol must also be provided in the manuscript.

Field-specific reporting

Please select the one below that is the best fit for your research. If you are not sure, read the appropriate sections before making your selection.

[x] Life sciences [ ] Behavioural & social sciences [ ] Ecological, evolutionary & environmental sciences

For a reference copy of the document with all sections, see nature.com/documents/nr-reporting-summary-flat.pdf

Life sciences study design

All studies must disclose on these points even when the disclosure is negative.

Sample size	Sample sizes were selected based on established standards for in vitro cells experiments and prior publications using HiPSCs-derived RPE. Allocation of sample to experimental groups was performed randomly. No formal statistical sample size calculation was performed. The number of biological and technical replicates was sufficient to detect reproducible effects across experiments.
Data exclusions	No data were excluded from the analyses unless predefined quality-control criteria were not met (e.g., image acquisition artefacts or cell death unrelated to experimental conditions). All exclusion criteria were pre-established.
Replication	All key findings were reproduced in at least three independent biological replicates.
Randomization	Blinding was not relevant to this study because sample identity could be objectively determined by experimental condition and automated image-based analysis pipelines were used where applicable.
Blinding	Blinding was not relevant to this study because sample identity could be objectively determined by experimental condition and automated image-based analysis pipelines were used where applicable.

Reporting for specific materials, systems and methods

We require information from authors about some types of materials, experimental systems and methods used in many studies. Here, indicate whether each material, system or method listed is relevant to your study. If you are not sure if a list item applies to your research, read the appropriate section before selecting a response.

Materials & experimental systems

n/a	Involved in the study	
[ ]	[x]	Antibodies
[ ]	[x]	Eukaryotic cell lines
[x]	[ ]	Palaeontology and archaeology
[x]	[ ]	Animals and other organisms
[x]	[ ]	Clinical data
[x]	[ ]	Dual use research of concern
[x]	[ ]	Plants

Methods

n/a	Involved in the study	
[x]	[ ]	ChIP-seq
[x]	[ ]	Flow cytometry
[x]	[ ]	MRI-based neuroimaging

Antibodies

Antibodies used

The following primary antibodies were used in this study: phospho-myosin light chain 2 (Ser19) (Cell Signalling Technology, Cat# 3671) at 1:50 for immunofluorescence (IF) and at 1:1000 for western blot (WB), vinculin (Sigma-Aldrich, Cat# V4139) at 15 µg/ml (IF), ezrin (Abcam, Cat# ab4069) at 10 µg/ml (IF) and 1 µg/ml (WB), phospho-ezrin (Thr567) (Invitrogen, Cat# PA5-37763) at 5 µg/ml (IF) and 1 µg/ml (WB), and non-muscle myosin heavy chain IIB (BioLegend, Cat# 909901) at 4 µg/ml (IF).
Secondary antibodies included: Alexa Fluor 488 anti-mouse IgG (H+L) (Invitrogen, Cat# A11001) at 8 µg/ml (IF), Alexa Fluor 594 anti-mouse IgG (H+L) (Invitrogen, Cat# A11005) at 8 µg/ml (IF), Alexa Fluor 594 anti-rabbit IgG (H+L) (Invitrogen, Cat# A11012) at 8 µg/ml (IF), Alexa Fluor 647 anti-rabbit IgG (H+L) (Jackson ImmunoResearch/Dianova, Cat# 111-605-144) at 7.5 µg/ml (IF), Alexa Fluor 488 anti-rabbit IgG (H+L) (Jackson ImmunoResearch/Dianova, Cat# 711-546-152) at 8 µg/ml (IF), HRP anti-rabbit IgG (Abcam, Cat# ab99696) at 1:5000 (WB), and HRP anti-mouse IgG (H+L) (Jackson ImmunoResearch/Dianova, Cat# 115-035-003) at 1:5000 (WB).

Validation

All primary antibodies have been validated by the manufacturers for use in human cells and the specified applications (IF and/or WB). Secondary antibodies were validated by the suppliers for species-specific reactivity.

Eukaryotic cell lines

Policy information about cell lines and Sex and Gender in Research

Cell line source(s)

HiPSCs-derived RPE cells were obtained from Fujifilm Cellular Dynamics. Catalog number R1102

Authentication

Cell identity was authenticated by the provider (Fujifilm Cellular Dynamics) prior to distribution according to the supplier's quality control procedures.

Mycoplasma contamination

HiPSCs-derived RPE cells used in this study were routinely tested for mycoplasma contamination using a PCR-based detection kit. Testing was performed approximately once per month throughout the duration of the study, and no contamination was detected at any time.

Commonly misidentified lines
(See ICLAC register)

n/a

Plants

Seed stocks

n/a

Novel plant genotypes

n/a

Authentication

n/a
